# Perception of digital health in the Baltic Sea Region: insights of experts from nine countries

**DOI:** 10.1186/s12913-026-14065-5

**Published:** 2026-01-30

**Authors:** Nawroth Melissa, Hüttmann Nicola, Fleßa Steffen

**Affiliations:** 1 Lehrstuhl für Allgemeine Betriebswirtschaftslehre und Gesundheitsmanagement, Rechts- und Staatswissenschaftliche Fakultät, Universität Greifswald, Friedrich-Loeffler-Str. 70, D-17487 Greifswald, Germany; 2 Universitätsmedizin Greifswald, Fleischmannstraße 8, D-17489 Greifswald, Germany

**Keywords:** Baltic Sea Region, Data protection act, Digital health, Fragmentation, Innovation, Innovation adoption model, Telemedicine

## Abstract

**Background:**

Digital health has the potential to improve the effectiveness and efficiency of health care. However, its adoption in the countries of the Baltic Sea Region varies considerably. In order to improve the diffusion and speed of adoption of this innovation, it is necessary to know the barriers and enablers that improve or hinder the implementation of digital health.

**Methods:**

Based on an international workshop, we conducted guided interviews with 15 experts from 9 countries in the Baltic Sea Region to determine their perceptions of the use of the innovation, barriers to its adoption and enablers.

**Results:**

Structural factors such as national income or population density are perceived as less relevant. Instead, cultural values such as future orientation, risk-taking and trust are described as the most important factors in explaining the different rates of adoption between countries in the Baltic Sea Region. Important barriers to rapid adoption of digital health are also federal structures with a high degree of autonomy for regions, as well as a rather strict interpretation of data protection laws. Some interviewees emphasised the role of individuals who make digital health “their child”.

**Conclusions:**

The implementation of digital health depends less on economic conditions than on the commitment of policy makers to make it happen. Future developments, in particular artificial intelligence in healthcare, will require an even deeper penetration of digital health, which calls for urgent strategies to overcome the barriers to digital health.

**Supplementary Information:**

The online version contains supplementary material available at 10.1186/s12913-026-14065-5.

## Introduction

‘Digital health’ is a buzzword in twenty-first century healthcare, encompassing the entire process of healthcare digitalisation based on information and communication technologies (ICT), with the aim of improving healthcare as a whole (prevention, diagnosis, treatment, management, etc.) [1, 2]. The dissolution of the unity of place and action is a central element of digital healthcare. [3]. On the one hand, this could improve patient care by extending it to locations outside the service provider (e.g. hospital, doctor’s surgery). On the other hand, digital health could reduce the cost of traditional healthcare, for example by reducing travel expenses and concentrating scarce human and material resources they are most effective. [4]. This could lead to an increase in the efficiency of care [5, 6].

Digital health, as a new approach to modernising and improving healthcare, has already found its way into many countries. However, the status quo in the development and implementation of digital health varies. The Scandinavian countries of Denmark, Sweden, Finland, Norway and Estonia are at te top of the comparison. In contrast, Germany is in the lower midfield of several rankings [7]. This raises the question of which factors are driving the differences in the diffusion of digital health, and what advantages these leading countries may have over those that are lagging behind.

In the following, we want to analyse which factors influence the diffusion speed of digital health innovations in the Baltic Sea countries. In this context, innovation means any new idea, concept, methodology or product that is adopted by a certain group of users. It does not necessarily have to be the first time that it is attempted to apply it, but for a certain country or group it must be novel with the potential to become a new standard [8]. Furthermore, we use a broad concept of the term “digital health” as any application of information and communication technology in the healthcare sector [5] (see Table 1). For instance, the Digital Health Index (BDHI) of the Bertelsmann Foundation subsumes a broad set of concepts under this term, including E-Health, M-Health and telemedicine [3, 9].

**Table 1:** Dimensions of digital health [7]

Domain	Subdomain	Example
Infrastructure		Nationally unique patient identification number
		Nationally unique access regulations
		Provider and service register
		Technical data infrastructure
		Automatic reading of patient data
Legal framework		Data protection regulation
		Technical data security
		Technical standards
		Medical terminology, semantic standards
Institutional anchoring		National Digital Health Authority
		Financial resources and incentives
		Enforcement of standards
		Stakeholder engagement
Digital health applications	Electronic patient file	Medication list
		Patient short file
	Healthcare services	E-prescription
		Video consultations
		Appointment bookings
		Telehealth
	Healthcare information	Personal patient portal
		Health information portal
Health services research		Big data approaches

In the next section, we present the methodology of qualitative online interviews with experts from each Baltic Sea country. We then describe the results of the interviews. The paper concludes with a discussion of the findings in light of the literature and some conclusions.

## Methods

This research was conducted under the auspices of the Interdisciplinary Centre for Baltic Sea Region Research (IFZO) with the project “Diffusion of innovations in services of general interest using the example of healthcare”. Based on the experiences of an international workshop of “Think Rural in the Baltic Sea Region” in March 2023 [10]), we conducted guideline-based expert interviews to explore why the emergence and diffusion of digital health innovations in the healthcare sector vary so significantly in the countries of the Baltic Sea Region. The experts from Germany, Poland, Denmark, Sweden, Norway, Finland, Estonia, Latvia and Lithuania gave their assessment of the digital infrastructure, enabling factors, barriers and possible reasons for the different diffusion of innovations. Between June 2023 and August 2024, we prepared the study, identified and interviewed experts, and subsequently transcribed, analyzed, and interpreted the data.

The preparation of the interviews involved several steps. A semi-structured expert interview format was selected to allow both flexibility and openness during the conversations. The questions were based on the theory of innovation adoption with the main elements of innovation, diffusion, promotors, barriers and enablers [11]. The full guide is attached (see Attachment 1, full interview guide). We did not use a strict questionnaire in order to allow for deviations from the guideline based on the experts’ knowledge and sub-questions. The main questions (key questions) and variable sub-questions were grouped into three thematic blocks, including introductory questions (personal information, background information), a main block (digital infrastructure, barriers, promoters, diffusion) and concluding questions (comments). Based on the innovation model, the first author deducted the items from the respective model [11] and formulated the interview guideline (see attachment 1). The second author revised the guideline by stronger categorising and condensing the broad set of questions so that they could be answered within an interview of expected 60 minutes. All experts from the different countries were asked the same questions in order to obtain comparable results. The interviews were scheduled to last approximately 45 minutes.

The next step was to identify suitable interviewees with expertise in digital health in the healthcare sector in their own country. An international, DFG-funded workshop on “Innovations in the Baltic Sea Region” was held at the University of Greifswald in March 2023, bringing together experts on innovation and healthcare from all the Baltic Sea countries. Some of them - but not all - were also experts in digital health in their countries and of high relevance for this research. The research questions of the interview guidelines were discussed with them as a measure of quality control of the interviews to follow. Furthermore, the authors asked the participants of this conference to identify the respective digital health experts in their countries. We also carried out an online contact search. We also used a snowball system, naming other potential contacts in the interviews or asking them by email.

It would have been ideal to have at least interview partners from each country coming from academic, healthcare industry, government and civil society to cover all relevant perspectives. However, the sampling methodology resulted in a strong focus on academia and healthcare professionals as the participants of the workshop had a bias in this direction. In addition, it took much longer than expected to get at least a sufficient number of interview partners so that only 15 interviews could be done. The interview experts were, however, highly professional covering also different roles, including as members of the society.

In addition, potential contacts from a previous survey conducted as part of the project were screened and researched for expertise and availability [12]. Potential interviewees were sent personalised cover letters by email, together with an abridged version of the interview guide, which they were asked to review the material in advance in order to check that the content matched their own expertise and to prepare themselves. If no response was received, up to two follow-up reminders were sent at intervals of approximately 2–4 weeks. Once participation was confirmed, an interview date was arranged and the consent form was provided for signature. After each interview the authors discussed whether the saturation point was reached, i.e., whether the response was sufficient to provide an overview of the current situation in the country and collecting more data would most likely not yield new or relevant information. If not, the first and second author decided together to interview a second (or third) interviewee from that country. In all cases, we still had other potential interview partners on our list. In this way, a total of 15 interviews were conducted in the countries of the Baltic Sea region (see Table 2), out of approximately 60 potential contacts requested. All interviews were conducted digitally using the licensed Zoom platform.

**Table 2:** Interviewees

Country	Interviewees	Profession	Position	Focus
Germany	1	physician	CEO hospital chain	innovative technologies in emergency medicine
Poland	2	economist, physician	professors	e-Health, mobile health, communications
Denmark	2	sociology, engineer	professor	health informatics, health technology assessment
Sweden	1	pharmacist	Swedish eHealth Agency	coordination of digital health
Finland	3	manager, manager, IT specialist	management Institute for Health and Welfare; professors	implementation, health informatics, usability
Norway	1	IT specialist	professor	health informatics
Estonia	2	social scientist, pharmacist	professor, senior researcher	healthcare-networks and IT; social impact
Lithuania	1	public health	professor	impact of digital health on population
Latvia	2	manager, IT specialist	management companies	business analytics, technology assessment

As the table shows, we conducted 15 interviews with 9 stakeholders from academia and 6 from healthcare while some interviewees had overlapping responsibilities, e.g. working in academic and for the government at the same time. However, no member of the civil society was included.

We did not instruct the interviewees on a strict definition of digital health but asked them to use their own concept of this term. As most of them were from the academic field, they were quite aware of the broad set of terms subsumed under digital health. However, it was also the intention to give them the liberty to use their own concepts as long as they should explain them.

Once the interviews had been conducted, the content of the data collected was analysed. The qualitative content analysis method according to Philipp Mayring was chosen following the deductive category assignment strategy [13]. As the interview guideline was based on innovation theory, the category system with main categories and subcategories could be based on the interview guideline, and the categories were pre-defined for better categorisation (see Attachment 2 Codebook). The codebook contains six main categories (plus ‘other’ if no categorisation is possible), each with 2–4 subcategories for further subdivision. Once the transcripts had been made from the audio files, the text passages were analysed and classified into the categories using MAXQDA [14] and colour coding for differentiation. Based on the assessment of the researchers, there was no need for revising categories or coding guidelines as the results were quite consistent.

The total duration of the interviews ranged from approximately 27 minutes to 1 hour and 11 minutes. Using the colour coding, the text passages from the different interviews were summarised and listed under the corresponding categories and sub-categories. The content of the text passages was then summarised and analysed by country.

## Results

### Overview

In a first set of interview questions, respondents were asked to describe the digital health situation in their country and to identify strengths and weaknesses. A first dimension of digital health was the homogeneity of diffusion of the innovation across the country and population. Several respondents see a rural-urban divide, with much greater penetration in urban areas, although it is generally felt that digital health is (even more) beneficial to rural areas.

This urban-rural divide is closely connected to regional disparities. Many respondents noted that digital health is used differently across states, provinces or regions. This is partly due to political differences. The more autonomous these political units are, the more likely it is that variation will emerge in the adoption of digital health across regions.

A further differentiation was made in terms of age groups, i.e. that older people have greater difficulties in using and benefiting from digital health systems than younger people. This was seen as a consequence of digital literacy beyond health. Older people have less knowledge to use digital services and need more personal contact, whether they use digital banking or video consultations with their doctor.

In addition, interviewees described the digital infrastructure in their countries. This includes different dimensions, such as the availability of general IT, networks and connectivity (e.g. quality of WIFI), but also specific dimensions for the healthcare sector, such as connectivity of mobile services (e.g. telemedicine emergency services), e-prescription, digital patient records, etc. Based on the interviews, the countries started at the same time to implement digital health with effort. For instance, Estonia and Finland started the digital health initiative 20–25 years ago and with admirable speed. Thus, digital health has become a standard in these countries by now. Other countries, like Germany or Poland, started much later. According to the interview partners, the speed of adoption the digital health innovation was not high enough to make good for this late take-off so that they are still lagging behind. A soft form of infrastructure is the population’s knowledge and ability to use digital services. As noted above, there are large differences between and within countries.

**Fig. 1:**
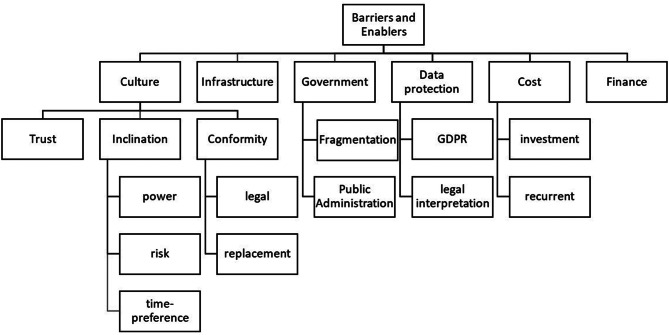
Barriers and enablers of digital health innovation. Source: own

Figure 1 shows the barriers and enablers discussed by respondents. In most cases, barriers and enablers are two sides of the same factor. For instance, a high degree of trust is an enabler of innovation adoption, a low degree of trust is a barrier against innovation adoption. In most cases, the direction of influence is clear, e.g. better finance is an enabler, shortage of funds a barrier. In the following, we will, thus, only explain the factors as barriers assuming that the opposite is appropriate as enablers.

Poor digital infrastructure, insufficient funds, high investment cost and recurrent costs are barriers to the implementation and uptake of digital health in a country. However, the interviewees referred to good infrastructure as enabler or poor infrastructure as barrier several times, but they did not focus strongly on the economic dimension. Only the experts from Latvia put some emphasis on it. Instead, respondents focused more on aspects beyond traditional economics, in particular a country’s culture. Individuals, groups and nations differ in their time preference, i.e. short-term thinking, financing and planning will lead to a reduced speed of implementation of digital health, as this macro-innovation cannot be implemented in a short-term project, but requires a long-term commitment at all levels. The more future-oriented an individual or culture is, the more likely it is to develop a propensity for innovation and change.

Risk aversion is another cultural dimension that can become a barrier to the uptake of digital health. Changing an existing and functional system to a new and unfamiliar one carries risks. The less people appreciate risk, the less likely they are to support digital health innovation.

Some interviewees also said that digital health involves a power game between different levels of government. The more people insist on their power, the less they will delegate and the less they will value digital health that empowers local providers and patients. Power distance, risk aversion and time preference determine the propensity of an individual, group or culture to promote digital health. The interviewees mentioned these barriers several times.

Another cultural dimension is conformity, which is “the tendency for an individual to align their attitudes, beliefs, and behaviours with those of the people around them. Conformity can take the form of overt social pressure or subtler, unconscious influence” [15]. In our context, this can have two dimensions. Legal conformity means that people tend to follow laws and regulations. For example, if a government decides to make the use of digital health mandatory, conforming cultures will follow this command and support the implementation of digital health. Here, conformity refers to in particular to the general public and healthcare professionals affected by certain laws and regulations. Conformity can also mean that people want to be consistent with the past and not make major changes to processes or traditions. “We do what we have always done” is a major barrier to innovation. This is relevant for all stakeholders, i.e. general public, academia, healthcare professionals and government officials.

Implementing digital health as a new technology also requires trust in healthcare providers, IT services, government and life in general. The less trust people have in institutions, the less likely they are to entrust their data to anonymous processes such as the internet. Countries are trying to increase trust in digital health by building strong institutions (e.g. e-services and innovation department, Estonia 2014) [16, 17]). The General Data Protection Regulation (GDPR) is seen by interview partners with a strong academic and/or professional background as a tool to build trust that all data will be professionally protected. At the same time, some interviewees try to reflect on the perception of the regulation by the general public seeing the regulation as an obstacle because the strict data protection rules make the transfer and use of data for medical decisions and research very cumbersome. Others argue that the same GDPR is interpreted quite differently in different countries, in particular by government officials. It is mandatory for all EU member states, as well as Norway as a member of EFTA (European Free Trade Association), but its application depends heavily on national interpretation. In some cases, the GDPR is seen as a driver of trust and thus innovation, while in other countries it is more of an obstacle to the spread of digital health.

Finally, the capacity of the government is perceived by some interviewees as a factor influencing the adoption of the digital health innovation. Poor administration can be a barrier to innovation, but also fragmentation. Public administration of smaller countries, such as Estonia, is rather centralized, while larger countries, such as Germany, are either divided into states that act quite independently or services ad decentralized on lower levels. In more centralized countries, digital health innovation can be implemented from the top down across the country, but in a fragmentated system, the successful implementation of digital health in one administrative unit (e.g. state) does not guarantee that other administrative units will follow. In this way, fragmentation or federalism can become a barrier to digital health innovation.

### Country situation

In the following section, we analyse the situation in different countries and explain the main features of each country in more detail. Table 3 shows the main barriers for different countries.

**Table 3:** Barriers in different countries based on interviews. Triangulation source [18, 19]

Country	Time- preference	Risk	Power	Trust	Fragmentation	Data protection
Germany	high	averse	hierarchy relevant	middle	high	barrier
Poland	high	very averse	hierarchy important	low	low	barrier
Denmark	middle	risk seeking	expert power	important	-	main barrier
Sweden	high	risk seeking	less important	highly important	regional autonomy	no barrier
Finland	low	averse	less important	highly important	rather low	barrier
Norway	middle	neutral	less important	highly important	-	barrier
Estonia	middle	neutral	less important	important	low	enabler
Lithuania	low	averse	hierarchy relevant	important	low	barrier
Latvia	low	averse	hierarchy relevant	highly important	low	enabler

#### Germany

The interviewee describes digital health innovation in Germany as a patchwork that varies from region to region and state to state. Similarly, rural and urban areas are very different, with examples of both very good and very poor digital infrastructure. A number of telemedicine applications are regularly used, but digital health is not yet standard and the pace of development is considered slow.

A major barrier according to the interviewees seems to be the lack of long-term funding, i.e. digital health projects are usually funded for a short period of time (e.g. 2 years), which does not allow them to mature and become the new standard. Even if a project is very successful, German federalism may prevent innovations from being adopted in other states.

The GDPR itself is not seen as problematic by the interview partner; the problem is rather the implementation with a strong dominance of the state data protection authorities. The GDPR sets the framework within which to operate, but the individual states within Germany interpret the regulation quite differently.

*“I perceive the General Data Protection Regulation as unsuspicious, because other countries have that too and they get things done. So, the problem is actually more at the level of state data protection authorities. In […* state given*], for example, we have a much more restrictive solution compared to others in Germany. I therefore believe that the GDPR sets the framework with which one must act, and that other (also EU) nations show that it is possible to work with it. But we make it so strict” (CEO hospital chain, Germany)*

In comparison to other country experts, the interviewee did not focus strongly on cultural values as barriers or enablers of the digital healthcare innovation adoption in Germany. However, the key informant confirmed that Germans are comparatively risk averse which might have a relevance for the adoption speed and degree of penetration as well.

#### Poland

The experts point to a weak digital infrastructure, but even more importantly, they highlight a low propensity to adopt digital health innovation, resulting in, among other things, a strong urban-rural divide. There are some “early adopters” in cities, but the majority of the rural population is reluctant to use digital health services due to a general aversion to new technologies and attitudes.

Poland has implemented a number of programmes to implement digital health solutions, particularly in suburban areas, many of them funded by EU projects. However, the interview partners state that majority of projects come to an end when their financial support comes to an end. The interview partners took mainly the perspective of their academic and/or healthcare professional background, but they also tried to take the position of the general public and government. They stated that the GDPR is seen as an obstacle in all stakeholder groups because it is perceived as too strict. In general, the experts consider their own culture to be comparatively slow to adopt innovations, including a high time preference and strong risk aversion. The orientation towards the present (“We appreciate today more than the future”) is seen by them as a barrier, while a high appreciation of future benefits is perceived as an enabler. In economic terms, a high time preference (short-term orientation) is a barrier, a low time-preference (long-time orientation) is an enabler [20]. Conversely, the centralism of the Polish government system could support the spread of digital health across the country once it is centrally decided. But so far there are not enough promoters.

*“But the problem is with attitudes and beliefs. People are traditional in their thinking. […] a lot of doctors, not only in primary healthcare but in general, are against, for example, teleconsultations. According to their beliefs, it’s not a good option” (physician and e-health specialist, Poland)*

#### Denmark

The digital health revolution started quite early in Denmark and has penetrated most areas of digital health. Many standards have now been developed and digital health has become routine throughout the country. In addition to sufficient budgets, respondents see a strong culture of innovation and rapid adoption of change as the main reasons for digital success. This also includes a “general sense of trust” among the majority of the population.

Although the Scandinavian countries are generally considered to be more egalitarian, the experts noted “power games” (as they called them). Digital health seems to take power away from an individual doctor and distribute it to a network of doctors and/or IT. This also implies a shift of power from individual doctors to networks or other professions.

These two interviewees point out that digital health implies a change in workflow, which requires further training, especially for doctors. At the same time, they also realise a number of digital health projects (“projectitis”) without systematic translation into routine care. Thus, they point out that there are still some shortcomings - but at a generally satisfactory level.

*“Why is it so difficult to lift these projects or these services from a project level to a daily routine level? I mean that we often call it “projectitis.” I mean, it’s like a disease where you’re sort of stuck in the project area. But also, I mean if you’re against some of these initiatives, it’s very easy to limit them by keeping them at a project level and not getting them into daily routine. Because as soon as they get into daily routine, it’s very difficult to get rid of them again”. (professor of health informatics, Denmark)*

#### Sweden

Sweden started quite early and now has a good digital infrastructure with a wide range of digital services that enable health data sharing. However, there are some regional disparities and the overall speed of innovation adoption could be better. This is partly due to the relatively high degree of independence of municipalities and regions, which is an obstacle to rapid, nationwide diffusion of digital health innovations. Currently, healthcare in Sweden is decentralised, i.e. the responsibility for implementing the digital health innovation lies with the 21 regional and 290 municipal councils.

The interviewee points to the same barrier as the colleagues from Germany and Denmark: short-term planning, funding and projects lead to a multitude of pilots without transfer into routines and standards. He calls this phenomenon “piloticities”. The gap between pilot and routine seems difficult to bridge, and the step from successful implementation in one region to another is even more difficult.

*“It’s a lot easier to do pilots and then to actually implement something; we’re not the only one or only country that really suffers from we used to call it “piloticities”. It’s like inflammation of pilots. We do pilots and nothing happens. It’s like 100 pilots and 95 of them is never implemented, which is sad because it´s a lot of good work being implemented, which is sad because it´s a lot of good work being not taking care of. Some of them are very good, so they should be taken care of”*. (coordinator of eHealth Agency, Sweden)

#### Finland

The three experts from Finland agreed that digital health is quite advanced in the country. Although the country is huge and has areas with very low population density, the distribution of digital health services seems to be quite even. However, there is an age gap, with older people less likely to use digital health services.

They also say that Finns are very interested in any kind of innovation, and they do not make differences between certain groups (e.g. general public, healthcare professionals, political leadership, or institutional stakeholders). All are said to be forward-looking and have no problem sharing power. Trust is of paramount importance to the Finnish population - one interviewee expressed this with the Finnish “habit” of leaving doors unlocked when leaving the house. They trust their fellow citizens, the government and their health services, including the responsible use of their health data. Mistrust – as expressed in the GDPR – is seen as inappropriate, so the implementation of the GDPR faces some resistance, albeit with different arguments than in countries like Germany.

The experts describe Finland as a country with high legal conformity of all population groups, i.e., the population as well as decision-makers in healthcare and government tend to follow the regulations and laws of higher levels and trust the goodwill of their leaders. At the same time, the experts are convinced that the general public and decision-makers might have a notion of “replacement conformity” mentioned in section “Overview”.

*“I would say in Finland our culture is so that we trust a lot on different kind of rules and regulations and things. […] I think in practice we are kind of following very strictly on those. So maybe that is something why it reflects in a way that those are hindering things and also leading to additional work-tasks instead of protecting something that is needed”. (professor of computer science, Finland)*

It was mentioned that Finland is perhaps the only country in the Baltic Sea region with two official languages (Swedish and Finnish). According to the interviewees, this has implications for all software, but it is a common challenge beyond the healthcare system.

#### Norway

Norway is the richest country in the region with a well-functioning IT infrastructure throughout the country, including fibre and wireless networks. Norsk Helsenett (Norwegian Health Network) is owned by the government and provides patient portals for every citizen.

According to the interviewee, Norwegian culture encourages innovation, i.e. Norwegians tend to be forward-looking, not too afraid of risk and have no problems sharing power. Trust is very important and includes trust in fellow citizens as well as in the government. Again, the interview partner saw these cultural strains relevant to all stakeholders. Compared to Sweden, Norway is a rather centralised country, i.e. the central government has managerial and financial responsibility for the health sector. There are four regional health authorities (out of five regions in Norway) that work closely with the central government.

*“It’s a bit hard for me to give you a consist answer. The biggest thing to learn from Norway and other Nordic countries, I think is that we really trust” (professor of informatics, Norway)*

However, the expert considers that there is still room for improvement, especially in the usability of digital tools. Furthermore, the implementation of the GDPR is seen by the expert as too strict, although the regulation itself is accepted. The expert is convinced that the regulation would give more freedom than the interpretation of local authorities.

The Norwegian interviewee was the only expert to mention digital health as a business case, i.e. Norwegian companies could offer services (e.g. reading radiology images) as a paid service globally.

#### Estonia

Estonia is considered to be at the forefront of digitalisation in Europe, and the two experts confirm that digital health is quite advanced. The government “owns” digitalisation, with a long line of prime ministers making digitalisation “their child”. Many services such as digital patient records, lifelong ID, e-prescription, etc. are standard and routine in Estonia.

According to the experts, the reasons for Estonia’s leading position in Europe are similar to those in other countries: a high level of innovation based on a forward-looking attitude, a willingness to take risks and trust of all stakeholders. This may not fully explain why Estonia is more advanced than neighbouring countries such as Latvia and Lithuania. However, respondents stressed that Estonia was simply the first country to put all its eggs in the digital basket. They started earlier, based on a very high level of commitment from the country’s leadership, including a ‘personal data protection law’ that is said to be stricter than the GDPR. The Estonian experts said that they see the data protection regulation as an enabler of digital health, not an obstacle, because it increases trust in the system. This statement – according to them – is not only true for academic and professional stakeholders, but also for politicians and civil society.

*“… the only particularity and what do we have in Estonia compared to other countries is that most users of healthcare and of digital systems, like everyday people, they trust. They trust these systems have been controlled well and, perhaps, they have less worries especially about data protection. I just trust […] the healthcare system in place that it is trustworthy and it’s secure“(researcher, Estonia).*

#### Lithuania

Lithuania has made some progress in digital health, but the pace of innovation seems to be slower than in Scandinavia or Estonia. Internet access and in particular the nationwide eHealth platform with access for the population are seen as encouraging factors. The government seems committed to the development of digital health and people trust that their data is well protected. However, the Lithuanian expert recognises a number of barriers to the implementation of digital health in his country. First, he notes that the development of the e-health system has been done without the participation of providers and patients, resulting in low usability and complaints that its use increases the administrative workload of healthcare providers.

Secondly, the propensity to innovate seems to be limited by risk aversion, in particular in the general population and institutional stakeholders. As in most settings, the likelihood of adopting digital health innovations decreases with age, but the expert points out that this tendency is even stronger in Lithuania. In contrast to risk aversion, time preference is low, which should lead to better adoption of innovations.

Thirdly, the Lithuanian expert is one of the few to point out the disparity between urban and rural areas, i.e. digital health is much more advanced in cities and towns than in villages. This, he argues, may be due to the government’s greater emphasis on urban development.

*It’s very big this difference [between urban and rural healthcare, question], mostly accessibility of specialists; general practise and PHC frame work quite OK. Though, when we’re speaking about rural, as rural villages surely with the previous system with the medical post, some of the settings were destroyed. […] I would say the problem of access to specialist is huge. […] We have no practise patient provider video consultation; everything is on telephone. […] So, there is no, let´s say, culture of good communication and it´s very big difference between these levels, ambitions and prestige and so one”*(professor and healthcare professional, Lithuania)

Fourth, the interpretation of the GDPR seems to be stricter in Lithuania than in other Baltic states. As he notes:

*“They use data protection mostly to protect themselves*”,

i.e. the GDPR is used as an argument to hinder digital health if it is not desired by certain stakeholders in particular of the civil society, but also from healthcare professionals and politicians.

#### Latvia

Latvia shares similar characteristics with Lithuania. However, data protection is not perceived as an obstacle by the experts. Both agree that Latvia has a good digital infrastructure, but standardisation of health data could be improved to better leverage interdependencies between sub-systems. As a small country, even cross-border data exchange is considered relevant, but language and semantic standards are a barrier.

They also agree that the majority of people are rather conservative and tend to avoid risks and stick to the “good old standards”. One of the experts talks about “a negative attitude towards any change”, especially among older people. Again, no discrimination between stakeholder groups is made.

*“I would say they [clinics for foreigners] are also likely to be the early adopters for any new innovation regarding digital health, but majority is very conservative as customers and so they will be difficult to persuade […] If we talk about patients, if they are open or reluctant, I would say it’s a generational thing. For instance, Gen. Z who are used to, you know just using messengers instead of calling as a way of Communicating, I would say they would be happy, family doctor and everybody else would be communicating with them not in their face to face, but also in messengers. But for majority it’s still the question of trust to devices and e-health as a way how to solve those problems which are related to personal health” (management scientist, Latvia)*

Only few mentioned financial constraints as a main problem, but the Latvian experts stressed independently that the government is not investing enough in the development of eHealth infrastructure. However, there is hope that this will change with a very enthusiastic and innovative new Minister of Health who has a strong focus on digital health. The new and comparably young minister (born 1974) took office in 2023 and, according to interviewees, has already made important contributions to the penetration of digital health in the health sector. In particular, he has developed a digital health strategy. However, they also state that the existing regulation demotivates the leaders of healthcare institutions to get more involved in digital health because they are afraid of breaking the rules. Latvians of all stakeholder groups seem to follow the regulations very strictly. For example, teleconsultation is rarely used for fear of being seen as unprofessional or violating data confidentiality laws. There were some efforts in this direction during the Covid-19 pandemic, but much was not continued afterwards.

## Discussion

The perception of digital health differs between the countries in the Baltic Sea Region. The results of this survey are consistent with the findings in the literature. For example, the Bertelsmann Foundation calculated a Digital Health Index (DHI) in 2018 with 17 OECD countries, including Estonia, Denmark, Sweden, Germany and Poland [21]. As Flessa & Hübner demonstrate, “there is hardly any correlation between country statistics and the DHI” [11], i.e. wealth, population, population density and health expenditure per capita do not determine the penetration of digital health in the health system in the respective countries. Instead, cultural values [22, 23] seem to be more important to understand the perception of digital health and the speed of innovation diffusion. They show that there is a “negative correlation between power distance and DHI, i.e., cultures with a strict and hierarchical leadership style have a lower penetration of the health care system with digital technologies. Likewise, cultures in which dominance, assertiveness or win-lose-thinking are seen as virtues (‘masculine cultures’ according to Geert Hofstede’s cultural dimensions [22, 23]) also tend to have a low DHI. The more people try to avoid uncertainty (and risks), the lower is the adoption of digital health” [11].

The” [24]” [24] also focusses on the digital innovation in the EU based on the digital intensity index (DESI) quantifying some aspects of digital health (e.g. access to e-health records). Within the Baltic Sea EU Countries, Denmark (score 98), Estonia (98), Lithuania (95) and Poland (90) are in the leading position, Germany (87), Latvia (85) and Finland (83) in the middle, Sweden (78) below the average of EU. Although these results differ from the self-perception of the experts, the recommendations for the countries are quite similar (e.g. data protection, empowerment of citizens and politicians, strategies etc. However, they also include the dimension of green transition which was hardly mentioned by our interview partners.

The results of this survey underline the findings of these different studies. The experts clearly show that there are differences between the nine countries. However, the fact that they ignored the relevance of financial resources or population size might indicate that these factors are not determining the digital health innovation adoption. It was shown that there is no significant correlation between the Bertelsmann Digital Health Index and these two factors [11, 25], we might also conclude that the experts did not focus on traditional economic aspects because they are not the determining factors. Instead, risk aversion, future orientation and willingness to change are much more important. Some of the interviewees emphasise that trust is an important determinant of perceptions of digital health. The role of trust in the willingness to accept change has been widely discussed in the literature, particularly regarding the right balance between trust and control. [26, 27]. Adoption of digital health innovations is not possible without confidence that the data will be confidential and properly protected. Since individuals cannot assess this protection themselves, trust in government and its administration is a prerequisite for the diffusion of digital health. Trust, as a cultural factor, differs from nation to nation and across historical trajectories.

However, even these cultural factors alone cannot explain the differences, which require further analysis. Interviewees highlighted the role of data protection legislation and its implementation. This aspect is also discussed by Sliwa et al. for the uptake of e-health in Germany, Austria and Denmark. They identify complexity and documentation requirements as major barriers (Austria, Germany) and practical government regulations as promoters (Denmark) of e-health [8]. Respondents to this survey also identified administrative barriers, but these are not necessarily the most significant. Instead, they emphasise the role of data protection regulation and implementation. There is still relatively little literature on the role of data protection laws and individuals’ willingness to share data in digital records as a barrier to digital health, but interviewees strongly emphasise that this may be key to understanding why digital health adoption varies so widely.

**Fig. 2:**
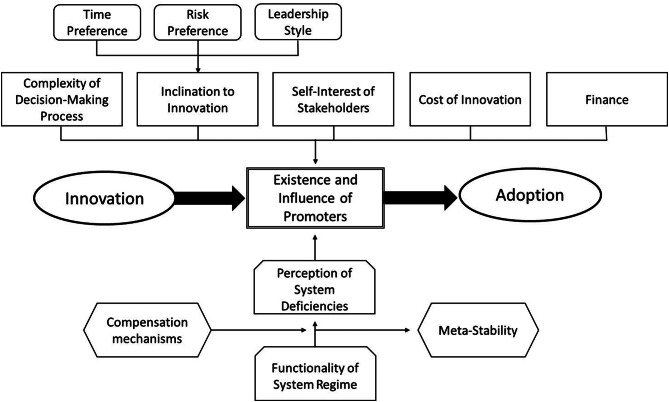
Model of adoption of healthcare innovations with barriers and enablers [11]

In summary, the model shown in Fig. 2 can help to understand the adoption of health innovations [11] by the promotor, i.e. the person or group of person supporting and facilitating the adoption of the innovation. It also allows the identification of barriers and enablers as well as their impact on the adoption process, and the appropriate placement of available tools to overcome them. Again, barriers and enablers are frequently the consequences of positive or negative factors. For instance, a high time preference constitutes a barrier, a low time preference an enabler of an innovation adoption.

The functionality of the existing standard of diagnosis and treatment as well as their administration is the starting point [28]. A fully functioning and stable system will not be changed, i.e. the digital health innovation will meet more resistance in countries where the health system works very well. However, even if there are perceived shortcomings in the current system, it is more likely that improvements will be made to it than that a radical innovation involving risks and costs will be introduced. These compensatory measures lead to artificial stability or metastability [29]. It is only when this stabilization is no longer sufficient that the pressure to find alternative solutions becomes dominant and the likelihood of adopting an innovation increases measurably. As a result, countries that had weak and difficult healthcare systems in the 1990s (especially the post-Soviet Baltic States) had a much higher chance of jumping on the digital health innovation than countries with well-established healthcare systems, such as Germany. However, meta-stability is no fate, as the example of Scandinavian countries demonstrate which had also quite well-working healthcare systems but still developed digital health early and rapidly. The enablers and barriers are tearing in different directions of success, and only the combination of all factors determines the final direction. It is likely that Finland, for instance, faced some meta-stability because the digital health innovation first had to overcome the stabile system regime. However, other factors, such as the culture and a rather early take-off surpassed this barrier.

Beyond that, the interviewees did not suggest any specific patterns among countries that have undergone post-communist transitions. One might expect that a common history of surveillance, political control, and institutional mistrust might still determine their willingness to accept innovative digital technologies, but the results of the respective countries are too diverse to draw any conclusions.

Furthermore, the ability and willingness to promote the adoption of an innovation depend on several factors, including the complexity of the decision. The more complex an innovation, the less likely it is to be adopted. Digital health requires a network of diverse providers, standardized processes and semantics. As such, it is an innovation that is likely to encounter resistance.

In addition, the propensity of stakeholders to innovate is crucial, depending on their time preference, individual risk preference and management approach. If people are future-oriented and risk-seeking, they are more likely to accept innovation. In addition, the management style within the organization influences the propensity to innovate. The more strict, hierarchical and dominant a leadership style is, the less likely it is that innovations will be developed and adopted [30–32]. Thus, countries with a very conservative leadership style are less likely to adopt digital health innovations.

The innovation adoption model can also be seen as the foundation of the analysis of the political process of different groups and perspectives. General public, healthcare professionals, political leadership, or institutional stakeholders have different incentives to adopt the digital health care innovation resulting in different penetration and speed of adoption. The interview partners point out that all groups can have “early knowers” or “laggards” resp. barriers and enablers of the digital health innovation [33, 34]. The transformation of the healthcare towards a fully digitalized sector is likely to follow an evolutionary reconfiguration process [35] resulting in fully digitalized niches while the entire landscape is still lagging behind. However, the speed of adoption of the innovation will depend on the power of these stakeholder groups.

There are a number of limitations to the findings presented in this study. Firstly, it is a qualitative study based on 15 interviews of nine countries. We selected experts mainly from academia (professors) and healthcare industry (e.g. CEO hospital chain), but the input from government officials and society was mainly based on the literature. It would be ideal to have at least 36 interview partners in order to capture different perceptions of stakeholder groups, i.e., one of each major stakeholder group per country. This would also safeguard that we could make a cross-country analysis based on stakeholder levels. Our experts came primarily from academia and/or healthcare profession. They were asked to take also the perspective of the civil society and government, but we are aware that this can be biased. However, due to limited resources this was not feasible to increase the sample of the extensive interviews.

The interviewees clearly point to differences between countries that are in line with the literature. However, with the existing methodology and sample, we cannot be completely sure whether these perceived differences are due to the selection of experts or to real country differences. Thus, our results suggest the need for further research with a broader pool of experts, including government officials and civil society.

Secondly, our work is based on the perception of key stakeholders, not on statistics, facts and figures. We recorded these perceptions because decisions of stakeholders are usually based on their assumptions, feelings and perceptions. However, our results call for a triangulation of official statistics and the perceptions stated in our paper.

Thirdly, our results are (as always) influenced by the choice of criteria and the respective interview guidelines (see Attachment 3). We tried to mitigate this limitation by using a semi-structured interview with open questions and sufficient time. The interviewees used this time to elaborate on a wide range of issues. However, we cannot be absolutely certain that there are no arguments beyond the bounded rationality of the researchers.

## Conclusions

The study shows that perceptions of digital health vary across the Baltic Sea Region. The most frequently mentioned barriers are cost, data protection laws, infrastructure, infrastructure and culture. Based on the interviews, we can say that the GDPR itself seems no to be the main barrier. It is the same for all countries, but its interpretation and implementation in the countries is different. Countries that are facing a slow adoption of digital health could consider relaxing their interpretation of this regulation. However, this must not jeopardise data protection, as this could lead to reduced trust and reservations about digital health. The Scandinavian countries and Estonia show that data can be protected without over-regulating data protection.

Fragmentation is another aspect that should be further analysed. Smaller and centralised countries have the advantage that an innovation can spread to all locations without further barriers. Countries with decentralised systems and/or more independent federal states find it more difficult to develop universal coverage of digital services across the country. The “here-not-invented” syndrome can block the diffusion of promising innovations from administrative unit to administrative unit or from state to state. Our interviews suggest that in Germany, for example, it would be helpful to shift some decision-making power on digital health from the states to the federal government.

Finally, the pace of innovation in digital health is influenced by cultural values such as trust, power distance, risk aversion, time preference and compliance. It is difficult to recommend changing cultural values, but governments can do a lot to build trust by demonstrating trustworthiness. Patient data must be strictly protected to enable patients to develop and accept lower levels of perceived risk.

Finally, respondents identified a number of individuals as key stakeholders in digital health innovation. It is recommended to support these “champions” who are inclined towards digital health. There is no doubt that future developments, in particular artificial intelligence in healthcare, will require even deeper penetration of digital health. There is an urgent need to overcome the barriers to digital health in some countries (e.g. Germany). Otherwise, they will fall further behind.

## Electronic supplementary material

Below is the link to the electronic supplementary material.

Supplementary Material 1

Supplementary Material 2

Supplementary Material 3

## Data Availability

The interview data utilized and analysed in this study can be obtained from the corresponding author upon request.
