# Supplementary material for: Perception of digital health in the Baltic Sea Region: insights of experts from nine countries
Source: BMC Health Serv Res. 2026 Jan 30;26:301. doi: 10.1186/s12913-026-14065-5 (PMC12930598; doi:10.1186/s12913-026-14065-5)
Supplement: Supplementary file 1 — Supplementary Material 1 [file 12913_2026_14065_MOESM1_ESM.docx]

# Attachment 1: Interview Guideline

Interview guide for the work package

*“Diffusion of innovations in services of general interest*

*using the example of health care”*

Interview: XX

Date: XX

Duration of the Interview: approx. 30-45 minutes

Introduction

- *Opening the conversation and introducing myself:*

I will shortly introduce myself: I am Melissa Nawroth, research assistant at the University of Greifswald and Interdisciplinary Research Centre for Baltic Sea Region Research. I am currently part of the project “Innovations and Policy Mobilities”, specifically working for the work package “Diffusion of innovations”.

- *Brief explanation of the research project:*

The interview takes place within the framework of the work package “Diffusion of innovations in services of general interest using the example of health care” and aims to identify barriers and promoters to the use of innovations in the countries of the Baltic Sea Region, particularly in rural areas.

…

- *Information about the interview procedure:*

The interview is expected to take 30-45 minutes of your time. The interview is divided into 3 sections, each comprising a maximum of 4 main questions including sub-questions for specification. It is possible to skip individual questions and continue the interview elsewhere.

- *Audio recording of the interview:*

By signing the document “Information and Declaration of Consent”, you give your consent to the recording of the interview for the purpose of transcription. Of course, both the audio recordings and the transcription of the interview will be treated confidentially and will not be passed on to unauthorised third parties.

- Before we start the interview, do you have any questions about the content or process of the interview or any other questions?

Personal information (introductory questions)

- Can you please briefly introduce yourself and state your profession?
- To what extent do you conduct research in the field of Electronic Health? (Emergency medical Service?)
- Do you have a special research focus in this area?

Main part

*Digital infrastructure*

- Would you say that your country has a **well-developed digital infrastructure**?
- *Yes, why? / No, why?*
- Interoperability? -meaning: different systems working together/functioning as one system
- Is there a **noticeable difference/disparity** in the digital infrastructure in **rural and urban areas**? / Internet access rural regions?
- Are the innovations in health care adapted to the special circumstances of rural areas in your country? (Internet connection, infrastructure, technical devices, for example offering video consultation because of low population density or infrastructure?)
- Is the **expansion** of the **digital infrastructure (in rural areas)** **supported by the government**? If so, how? (special funding for rural regions?)
- Are telemedicine/**eHealth applications** involved in **the day-to-day service** in healthcare? **(EHR, ePrescription, Video consultation and so on)**

*Barriers*

- Is there a **clear difference** in **health care provision in rural and urban areas** of your country noticeable?
- **Examples** of where the difference can be seen? (Limited access to healthcare facilities and providers, limited resources, restricted funding, shortage of physicians or other personnel, lack of acceptance/lack of use of telemedicine applications?)
- Do you know of **specific problems that exist in the provision of health care particularly in rural areas** of your country? Which? (details, examples)
- Could **they (Problems)** be remedied **(removed/eliminated) through the use of telemedicine applications**?
- In your opinion, what are the **main barriers to a successful implementation/ further development of Electronic Health innovations**/applications? (Emergency Medical Service?)
- (perhaps you can include the following points)
- **Within your country urban to rural**
- **Spread from one country to another**
- Do you know of **specific applications that are not used in your country** (or cannot be used in rural areas of your country), **perhaps because they are rejected by the authorities or providers?**
- Maybe you can **explain** it **using the example of Electronic Health Record** (do you have different versions in hospitals and are they working together, or is there just one?), ePrescription/eReferral/eSick Leave Certificate, video consultations, eConsults between providers
- Are there **regulations** in your country **for the use or implementation of eHealth applications** that **also affect standard healthcare service**?
- Like a **regulation/law only for eHealth applications**?
- Affect the standard healthcare provision: meaning: making it easier, supporting the day-to-day service in healthcare
- Are there **eHealth applications** in your country that are **used in healthcare but do not yet have a full legal basis?**
- Projects? If so, why?
- As you know, in the **European Union** we have the same **General Data Protection Regulation** of the European Union: Would you say the **data protection helps or hinders the use of eHealth applications in healthcare in your country**? (maybe using the example EHR, ePrescription etc.)

*Promoters*

- Does your country **provide funding to promote eHealth (EMS) innovations in rural areas**?
- Or is it more likely to be promoted in urban areas?
- Examples?
- Are there **other forms of support to drive telemedicine applications**? (networking, funded projects, ...)
- What **specific factors promote the use of eHealth applications** in your country? (or **persons**?)

*Diffusion*

- Can you think of **two eHealth applications whose use is very different** in your country and why?
- Different funding, acceptance, interoperability, technical problems, rural/urban disparity
- What do you think are **possible reasons why some eHealth applications**/innovations **do not find their way to other countries?**
- Perhaps you can **think of an eHealth (EMS) application that is used in another country but not yet in your own**, although it is known?
- Or can you think of a **specific application that is adopted from another country and now used in your country?**
- Would you say **eHealth innovations in health care are quickly accepted by providers and users** in your country?
- **EHR? ePrescription? eReferral? eSick Leave Certificate? Telemonitoring? Video Consultation provider-provider/ Provider-patient?, medical drone, tele-emergency physician, etc)**
- In your opinion, what **opportunities and risks arise from the use of eHealth in healthcare**?
- What are the advantages and disadvantages of using Electronic Health applications in healthcare in the **future**?

Closing questions

- We have now reached the end of the interview. Do you have the impression that important aspects were not addressed or would you like to add something?
- Thank you very much for your support, your time and the pleasant conversation.
- Discuss how to proceed, such as sending the transcripts for review.
