# Supplementary material for: Perception of digital health in the Baltic Sea Region: insights of experts from nine countries
Source: BMC Health Serv Res. 2026 Jan 30;26:301. doi: 10.1186/s12913-026-14065-5 (PMC12930598; doi:10.1186/s12913-026-14065-5)
Supplement: Supplementary file 2 — Supplementary Material 2 [file 12913_2026_14065_MOESM2_ESM.docx]

# Attachment 2: Codebook

Code Book for the work package

*“Diffusion of innovations in services of general interest using the example of health care”*

| Main category | Sub-category | Colour in document | Definition |
| --- | --- | --- | --- |
| General Information | profession | brown | General statements or background information about the interviewee and their work |
|  | expertise |  |  |
| Digital Infrastructure | Rural-urban distribution (or rather disparity) | blue | Information about the digital infrastructure; accessibility of the Internet and thus use of digital services in rural or urban areas; technical perspective; interoperability |
|  | Technical perspective |  |  |
| Barriers | Rural-urban difference | red | Obstacles in every respect (human resources, culture, technology, acceptance, ..) that prevent or impede the distribution and use of digital services (e-health applications) |
|  | Limited access to healthcare facilities and providers, limited resources, restricted funding, shortage of physicians or other personnel, lack of acceptance/lack of use of telemedicine applications |  |  |
| Regulation | Legal basis projects | orange | Legal framework conditions that influence the use, distribution and handling of digital services in a positive or negative way |
|  | General Data Protection Regulation |  |  |
|  | e-health law |  |  |
| Promoters | Funding (projects) | green | all factors or persons that support and promote the use, implementation or distribution of digital services in a positive way |
|  | Specific factors |  |  |
|  | Specific persons |  |  |
| Diffusion | Rural-urban difference | purple | everything that affects the diffusion of e-health applications between the different countries in any way |
|  | Diffusion of applications working/not working |  |  |
|  | International difference between countries |  |  |
|  | Adaption/adoption |  |  |
|  | Opportunities and risks future |  |  |
|  | Approval by providers and users |  |  |
| Other |  |  | Everything that cannot be assigned to the other categories |
