# Supplementary material for: Perception of digital health in the Baltic Sea Region: insights of experts from nine countries
Source: BMC Health Serv Res. 2026 Jan 30;26:301. doi: 10.1186/s12913-026-14065-5 (PMC12930598; doi:10.1186/s12913-026-14065-5)
Supplement: Supplementary file 3 — Supplementary Material 3 [file 12913_2026_14065_MOESM3_ESM.docx]

# Attachment 3: COREQ-Report

Consolidated criteria for reporting qualitative studies (COREQ) (Tong, Sainsbury et al. 2007)

| **No. Item** | **Guide questions/description** | **Reported on Page #** |
| --- | --- | --- |
| **Domain 1: Research team**  **and reﬂexivity** |  |  |
| *Personal Characteristics* |  |  |
| 1. Inter viewer/facilitator | Which author/s conducted the interview or focus group? | Author 1 with support of author 2 |
| 2. Credentials | What were the researcher’s credentials? (E.g. PhD, MD) | 1: MSc.  2: PhD |
| 3. Occupation | What was their occupation at the time of the study? | 1: research assistant  2: professor |
| 4. Gender | Was the researcher male or female? | 1: female  2: male |
| 5. Experience and training | What experience or training did the researcher have? | 1: 3 years post-graduate working experience in qualitative research  2: 35 years in quantitative and qualitative research |
| *Relationship with participants* |  |  |
| 6. Relationship established | Was a relationship established prior to study commencement? | Direct contact by mail. |
| 7. Participant knowledge of the interviewer | What did the participants know about the researcher? (e.g. personal goals, reasons for doing the research). | Letter of introduction sent before |
| 8. Interviewer characteristics | What characteristics were reported about the interviewer/facilitator? (e.g. Bias, assumptions, reasons and interests in the research topic) | Incl. explanation of wider research context |
| **Domain 2: Study design** |  |  |
| *Theoretical framework* |  |  |
| 9. Methodological orientation and Theory | What methodological orientation was stated to underpin the study? (e.g. grounded theory, discourse analysis, ethnography, phenomenology, content analysis). | Content analysis |
| *Participant selection* |  |  |
| 10. Sampling | How were participants selected? (e.g. purposive, convenience, consecutive, snowball) | Step 1: presence workshop with recommendations  Step 2: internet research  Step 2: snow ball method |
| 11. Method of approach | How were participants approached? (e.g. face- to-face, telephone, mail, email) | Video |
| 12. Sample size | How many participants were in the study? | 15 |
| 13. Non-participation | How many people refused to participate or dropped out? Reasons? | 45 |
| *Setting* |  |  |
| 14. Setting of data collection | Where was the data collected? (e.g. home, clinic, workplace) | Home, workplace |
| 15. Presence of non- participants | Was anyone else present besides the participants and researchers? | No |
| 16. Description of sample | What are the important characteristics of the sample? (e.g. demographic data, date) | See table 2 |
| *Data collection* |  |  |
| 17. Interview guide | Were questions, prompts, guides provided by the authors? Was it pilot tested? | Pilot test during workshop; guideline sent before |
| 18. Repeat interviews | Were repeat interviews carried out? If yes, how many? | No. |
| 19. Audio/visual recording | Did the research use audio or visual recording to collect the data? | Audio recording |
| 20. Field notes | Were ﬁeld notes made during and/or after the interview or focus group? | Audio recording; personal notes |
| 21. Duration | What was the duration of the inter views or focus group? | 27 minutes to 1 hour and 11 minutes. |
| 22. Data saturation | Was data saturation discussed? | Between researchers, for each country |
| 23. Transcripts returned | Were transcripts returned to participants for comment and/or correction? | Yes. |
| **Domain 3: analysis and ﬁndings** |  |  |
| *Data analysis* |  |  |
| 24. Number of data coders | How many data coders coded the data? | 1 |
| 25. Description of the coding tree | Did authors provide a description of the coding tree? | yes |
| 26. Derivation of themes | Were themes identiﬁed in advance or derived from the data? | In advance and compared |
| 27. Software | What software, if applicable, was used to manage the data? | MAXQDA |
| 28. Participant checking | Did participants provide feedback on the ﬁndings? | Had been asked, but no response |
| *Reporting* |  |  |
| 29. Quotations presented | Were participant quotations presented to illustrate the themes/ﬁndings? Was each  quotation identiﬁed? (e.g. participant number) | Identified with profession and country. |
